# Diphtheria and Its Relation to the Laboratory

**Published:** 1910-05

**Authors:** K. R. Collins

**Affiliations:** Atlanta, Ga.


### Journal-Record of Medicine

**Successor to Atlanta Medical and Surgical Journal, Established 1855, and Southern Medical Record, Established 1870.**

**OWNED BY THE ATLATNA MEDICAL JOURNAL CO.**

**Published Monthly.**

*Official Organ Fulton County Medical Society, State Examining Board, Presbyterian Hospital, Atlanta, Birmingham and Atlantic Railroad Surgeons' Association, Chattahoochee Valley Medical and Surgical Association, Etc.*

EDGAR G. BALLENGER, M. D., Editor.
BERNARD WOLFF, M. D., Supervising Editor.
A. W. STIRLING, M. D., C. M., D. P. H., J. S. HURT, B. Ph., M, D,
GEO. M. NILES, M. D., W. J. LOVE, M. D., (Ala.); Associate Editors.
E. W. ALLEN, Business Manager.

COLLABORATORS

DR. W. F. WESTMORLAND, General Surgery.
F. W. McRAE, M. D., Abdominal Surgery.
H. F. HARRIS, M. D., Pathology and Bacteriology.
E. B. BLOCK, M. D., Diseases of the Nervous System.
MICHEL HOKE, M. D., Orthopedic Surgery.
CYRUS W. STRICKLER, M. D., Legal Medicine and Medical Legislation.
E. C. DAVIS, A. B., M. D., Obstetrics.
E. G. JONES, A. B., M. D., Gynecology.
R. T. DORSEY, Jr., B. S. M. D., Medicine.
L. M. GAINES, A. B., M. D., Internal Medicine.
J. N. LeCONTE, M. D., Diseases of the Stomach and Intestines
L. B. CLARKE, M. D., Pediatrics.
EDGAR PAULIN, M. D., Opsonic Medicine.
R. R. DALY, M. D., Medical Society.
A. W. STIRLING, M. D., Etc., Diseases of the Eye, Ear, Nose and Throat.
BERNARD WOLFF, M. D., Diseases of the Skin.
E. G. BALLENGER, M. D., Diseases of the Genito Urinary Organs.

VOL. LVI. MAY, 1910 No. 2.

### DIPHTHERIA AND ITS RELATION TO THE LABORATORY.

K. R. COLLINS, M. D., ATLANTA, GA.

A disease corresponding in its description to diphtheria occurred in the earliest Babylonian days. No mention of such disease, however, is found in the history of the Greeks. Hence it is supposed to have been introduced into the European countries from Egypt. Much controversy arose during the 16th century concerning the pharyngeal type of the disease, especially its relation to membranous croup. This discussion took place principally in France, where the disease made great ravages. A

nephew of Napoleon, son of the King of Holland, died of croup and later the Empress Josephine. Napoleon offered a reward at different times to anyone who would establish the relation between these two diseases. Many observers attempted a solution of this problem, but it was not until 1821 when Bretonneau published an essay so complete in its observations that little was added until the diphtheria bacillus and its relation to the disease was established in 1883, first by Klebs and later confirmed by Loeffler. From this time on the therapy and etiology of the disease has been thoroughly studied, so that at the present day with a specific cure in our hands, an understanding of the etiology and the method by which it is spread, the disease is comparatively little to be dreaded excepting in a few instances. Prompt action in the administration of antitoxin early in the disease and rigorous methods resorted to for the prevention of contagion are the chief requisites to place it in the roll of amenable diseases.

The chief sources of infection are from human to human, or from articles of clothing, pencils, etc., that are used about a patient ill of this disease. School children are a frequent source of the spread of this disease from the fact that children with sore throats are allowed to go to school, and, as we all know, they are very careless in handling slate-pencils, books, slates, etc., of their companions. Where a supposed sore throat develops into diphtheria a great deal of harm has already been done before a true recognition of the disease occurs.

Human carriers are a factor to be dealt with in the spread of the disease. The question of quarantining a normal individual is a momentous one, and would be difficult to carry out. In the case of boarding schools every student should on return for the Fall term present a certificate of health stating that after thorough examination the individual is found to be free from bacillus of diphtheria, bacillus of typhoid fever and allied types, plsamodia of malaria, etc., as this would control to a certain extent outbreaks of such diseases in schools. In public schools children giving history of suffering from these diseases during vacations should be submitted in the same tests.

Milk is a frequent source of contagion where the disease occurs in a family of dairymen, among the milkers or others caring for the milk in any way, and carelessness on the part of individuals in cleaning bottles to be returned to the dairy from homes where the disease exists. Flies can carry the disease, but as the life of the diphtheria bacillus under such conditions is short, as will be stated later, the danger is not so great in this disease as in typhoid and other diseases of bacterial origin.

Guinea pigs, rabbits, chickens, pigeons, birds and cats are affected by the toxines from the diphtheria bacillus; dogs, goats, cattle and horses in a much less degree, while rats and mice are rarely affected. The disease has sometimes been conveyed by cats, possibly dogs, but the disease which develops frequently in animals is due to an organism similar to the diphtheria bacillus, but which cannot produce the diphtheria in man, and antitoxin has no effect upon such cases.

The bacillus persists in the throat after infection for varying periods of time, from three days after the disappearance of the membrane to nine weeks, according to Park and other authorities. Virulent bacilli may be found in the throats of healthy individuals who have been brought in contact with the disease. Such individuals are a source of danger to those who come in contact with them, not perhaps to such a great extent as the individual having the disease, on account of there being practically no discharge from the throat, but nevertheless it is perfectly possible for them to convey the disease to others. The diphtheria bacilli are readily killed by exposure to sunlight, but in membrane that remains moist they may remain alive for months. They have been found on slate pencils after some weeks, but on coins they die out in a few hours. They will live in milk at a temperature as low as 20 degrees without any effect on the milk.

Sore throats that are in the least suspicious demand a bacteriological examination. Where clinical evidences of diphtheria exist it is frequently advisable to give antitoxin without waiting for the results of this examination, but even then bacteriological examinations should be made, and a culture is taken if possible before the antitoxin is administered. This confirms the diagnosis, and I am sure that every physician will find it helpful to him in future cases. (I cannot urge too strongly the necessity of bacteriological examinations, as typical cases are frequently met with.) It is sometimes necessary to make more than one examination before the bacilli are found. This is particularly true of the laryngeal cases. There are instances where the bacilli under such conditions have never been obtained with a swab, but have been found in great numbers in the sputum. Careful examination of the ears is necessary in cases of diphtheria, as it is not unusual for the disease to be located here. The conjunctivas of the eye may be affected by the diphtheria bacillus, but this should not be confused with a condition occurring in this location, which is produced by the xerosis bacillus. While this organism resembles one of the pleomorphic forms of the diphtheria bacillus, morphologically, it is not related to it in a biochemical sense, and their toxins and antitoxins do not interact.

Health officers differ somewhat in the number of examinations to be made after recovery and before the release of the patient. Where the bacilli persist for a number of weeks or months after the disappearance of the membrane, the patience of the physician is often sorely tried by importunities on the part of the family or the individual being quarantined. Yet instances have occurred where, after several weeks, the patient has given one negative culture, been released, and diphtheria has developed in a member of the same family several days after his release. Upon re-examination the individual has shown the presence of diphtheria bacilli in the throat. In my opinion at least two consecutive negatives should be required before release of the patient.

The value of antitoxin in the treatment of diphtheria is, of course, beyond question. The mortality decreases in ratio to the time of administration with reference to the stage of the disease. The percentage of recovery where antitoxin is administered early and in sufficient quantities is high. Cases that are seen later in the establishment of the disease the chances diminish according to the severity and length of time that the disease has existed. Large doses of antitoxin given consecutively do not give untoward ersults. Where the results have been unfortunate the doses have often than otherwise been smaller than the first dose. Antitoxin has been given in quantities up to 100,000 units without bad results. Many symptoms occurring and after the administration of antitoxin have frequently erroneously been attributed to use of the antitoxin. Rashes may sometimes occur anywhere from a few hours to ten or twelve days after the administration of the serum. These rashes, while alarming to the family are harmless, and disappear within a few days, the discomfort being the only feature to be considered. In about one case in ten thousand the effect of antitoxin has been detrimental. It is generally conceded that three things may play a part in this; first, a person suffering from asthma may show marked cyanosis with collapse and death. This will happen with a small dose as well as a large dose, or the first dose administered. Sudden death has occurred after the administration of the first dose. This is supposed to be due to a condition known as status lymphaticus. This is a condition that is seldom recognized during life, and is of such rare occurrence that it need not be counted as a prominent factor in the contra-indication of the administration of antitoxin. The part that anaphylaxis may play in the use of this agent is not as yet a settled question, and I will not go into a discussion of it here, but will merely emphasize it as a point to be considered in the giving of antitoxin. The fatal effect of the first dose could readily be explained by a condition of what we might call normal anaphylaxis, corresponding to normal agglutins, normal immune bodies, etc. Just in what way the so-called normal anti-bodies are produced has never been satisfactorily explained. In the case of normal agglutins and normal immune bodies the specificity is not marked, but they are common to a type rather than peculiar to the individual. It is possible that in susceptible people the proteid equilibrium has been interfered with in such a way that either an analagous substance is produced and absorbed or split products, derived from an analagous proteid may have stimulated this hypersensitiveness in the individual, so that a first dose of any proteid belonging to a common type would call forth the same result.

It might be well to make the statement here for the benefit of those who are not already acquainted with the fact that the State Board of Health is prepared to send out diphtheria antitoxin for the use of the citizens of this state. It is provided by the legislature that the antitoxin be distributed through the Ordinaries of the counties, and application should be made to this office. The antitoxin is concentrated according to the Gibson method. This removes from the serum a number of proteids which are not concerned in the production of antitoxin, as the antitoxin is always found with the globulins of the serum. This is advantageous from the fact that it reduces the number of rashes and the concentration admits of a larger dose per bulk. It must be remembered, however, that concentration does not in any way reduce the possible dangers from anaphylaxis, as the proteids that are left in the serum, as well as the globulins, are quite capable of forming this substance in the blood. Every effort is made to insure to the State an efficient article. The tests, both as to strength and sterility, comply with the requirements established by the Marine Hospital.

